# A universal protocol for isolating retinal ON bipolar cells across species via fluorescence-activated cell sorting

**DOI:** 10.1016/j.omtm.2021.01.011

**Published:** 2021-01-26

**Authors:** Elisa Murenu, Marina Pavlou, Lisa Richter, Kleopatra Rapti, Sabrina Just, Jasmina Cehajic-Kapetanovic, Neda Tafrishi, Andrew Hayes, Rachel Scholey, Robert Lucas, Hildegard Büning, Dirk Grimm, Stylianos Michalakis

**Affiliations:** 1 Department of Ophthalmology, Ludwig-Maximilians-University Munich, 80336 Munich, Germany; 2 Department of Pharmacy, Ludwig-Maximilians-University Munich, 81377 Munich, Germany; 3 Core Facility Flow Cytometry, Biomedical Center, Ludwig-Maximilians-University Munich, 82152 Planegg-Martinsried, Germany; 4 Department of Infectious Diseases/Virology, Medical Faculty, University of Heidelberg, 69120 Heidelberg, Germany; 5 BioQuant Center, University of Heidelberg, 69120 Heidelberg, Germany; 6 Laboratory for Infection Biology and Gene Transfer, Institute of Experimental Hematology, Hannover Medical School, 30625 Hannover, Germany; 7 REBIRTH Research Center for Translational Regenerative Medicine, Hannover Medical School, 30625 Hannover, Germany; 8 Nuffield Laboratory of Ophthalmology, Nuffield Department of Clinical Neurosciences, Oxford University and Oxford University Hospitals, Oxford OX3 9DU, UK; 9 Core Facility Flow Cytometry, Gene Center, BioSysM, Ludwig-Maximilians-University Munich, 81377 Munich, Germany; 10 Center for Biological Timing & School of Biological Sciences, Faculty of Biology Medicine and Health, University of Manchester, Manchester M13 9PL, UK; 11 German Center for Infection Research (DZIF), partner site, Hannover, Germany; 12 German Center for Infection Research (DZIF) and German Center for Cardiovascular Research (DZHK), partner site, Heidelberg, Germany

**Keywords:** ON bipolar, FACS, AAV, retina, NHP, degeneration

## Abstract

Inherited retinal dystrophies (IRDs) are characterized by progressive degeneration and loss of light-sensing photoreceptors. The most promising therapeutic approach for IRDs is gene supplementation therapy using viral vectors, which requires the presence of viable photoreceptors at the time of intervention. At later disease stages, photoreceptors are lost and can no longer be rescued with this approach. For these patients, conferring light-sensing abilities to the remaining interneurons of the ON circuit (i.e., ON bipolar cells) using optogenetic tools poses an alternative treatment strategy. Such treatments, however, are hampered by the lack of efficient gene delivery tools targeting ON bipolar cells, which in turn rely on the effective isolation of these cells to facilitate tool development. Herein, we describe a method to selectively isolate ON bipolar cells via fluorescence-activated cell sorting (FACS), based on the expression of two intracellular markers. We show that the method is compatible with highly sensitive downstream analyses and suitable for the isolation of ON bipolar cells from healthy as well as degenerated mouse retinas. Moreover, we demonstrate that this approach works effectively using non-human primate (NHP) retinal tissue, thereby offering a reliable pipeline for universal screening strategies that do not require inter-species adaptations or transgenic animals.

## Graphical Abstract

**Figure:**
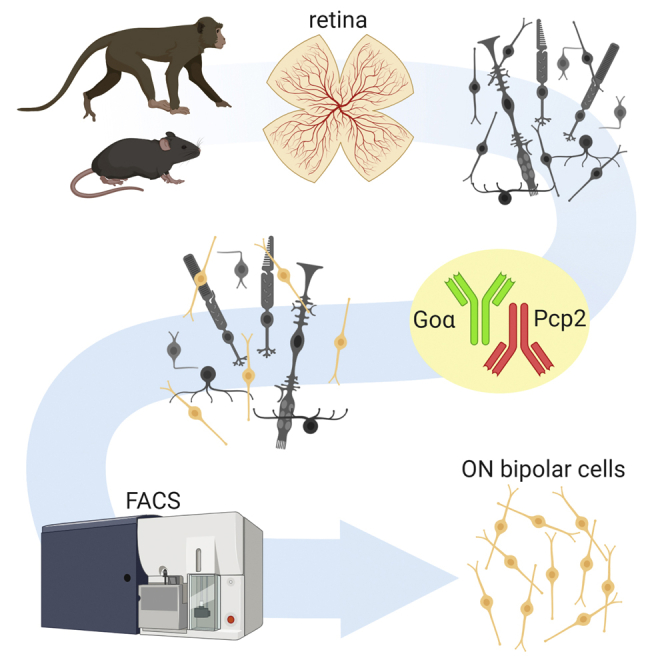

## Teaser

We describe a novel method to selectively isolate ON bipolar cells from mouse and non-human primate retinas. This method could enable the development of new tools that target the retinal ON circuit and lead to new therapies for inherited retinal dystrophies at late disease stages.

## Introduction

Inherited retinal dystrophies (IRDs) are a major cause of blindness worldwide. Among the approaches developed to alleviate this condition, gene supplementation therapy represents to date the most clinically relevant. By supplementing a mutated or missing gene in affected photoreceptors, it is possible to rescue their function and/or to halt their degeneration.^1^, ^2^, ^3^, ^4^ Although gene supplementation therapy is a promising approach, several limitations hamper its widespread application for the treatment of all IRD patients. More than 300 genes (https://sph.uth.edu/retnet) causing various forms of IRD are known and would require the development of as many “tailor-made” therapies to treat each of the affected patient populations. As gene supplementation therapy relies on the presence of viable and thus rescuable photoreceptors, only patients with early and mid-stage degeneration are amenable to such treatment. This requires the early diagnosis of patients via genetic testing, a process subject to further optimization. As such, new avenues are being explored to develop gene-independent, broadly applicable therapies that enable treatment at later disease stages, when no/almost no viable photoreceptors are left.^5^^,^^6^ Promising approaches focus on optogenetic tools delivered to ganglion cells, bipolar cells, or dormant (non-functional) cones in order to endow a previously blind retina with light sensitivity.^5^^,^^6^

The functional output of the mammalian retina is dictated by two overarching signaling circuits, the ON and OFF pathways, each relying on specific subtypes of neurons with distinct synaptic connectivity. For the purpose of restoring vision in diurnal mammals, targeting the ON circuit would match its natural capacity to respond to increasing brightness, as the OFF circuit responds predominantly to darkness.^7^^,^^8^ While ganglion cells are suitable candidates due to their accessible location, their position at the end of the retinal circuitry does not allow for any signal amplification or fine-tuning that is required for daylight vision. In this regard, ON bipolar cells are better suited targets for optogenetic therapies, as signals generated in these neurons are further processed within the retina at synapses in the inner plexiform layer (IPL)^8^ before they are sent to the thalamus by the ganglion cells. However, efficient vectors and tools that enable specific targeting of ON bipolar cells across species are still missing.

Indeed, ON bipolar cells have been the main target of optogenetic tool delivery with variable outcomes depending on the method. Different variants of the murine *Grm6* promoter (mGluR6)^9^, ^10^, ^11^, ^12^, ^13^, ^14^, ^15^, ^16^ and its human homolog^17^ have been explored to drive and optimally restrict gene expression to these cells, using applications such as *in vivo* electroporation^9^ with adjunctive therapies to enhance transduction,^18^ or engineered adeno-associated virus (AAV) vectors^10^^,^^19^ such as AAV2.Y444F^13^^,^^14^ and AAV2.7m8.^11^^,^^20^ Moreover, efforts to engineer AAVs that specifically target ON bipolar cells using directed evolution have been initiated.^10^ Nevertheless, all tool development approaches so far relied on transgenic mouse lines for screening, making it impossible to transpose these methods on animals evolutionarily closer to humans, e.g., non-human primates (NHPs). To facilitate specific viral or non-viral tool development, we aimed to establish a suitable method for efficient, selective, and cost-effective ON bipolar cell isolation across species. Importantly, such a broadly applicable ON bipolar cell isolation method could also help advance our understanding of ON bipolar cell biology in physiological as well as disease contexts.

To this end, we report herein a broadly applicable sorting protocol for isolating ON bipolar cells from healthy and degenerating retina models alike. We first established the method in mice and confirmed that our sorting strategy yields the enrichment of ON bipolar cells and, more importantly, the discrimination from OFF bipolar cells. Furthermore, we successfully implemented the protocol using NHP retinas, thereby demonstrating it is possible to specifically isolate ON bipolar cells in a species that better approximates human physiology. We propose this method as a basis for future investigations aiming to characterize ON bipolar cells as well as for the development of new tools to treat late-stage IRDs.

## Results

### Goα and Pcp2 label ON bipolar cells in healthy and degenerated mouse retinas

**Figure 1:**
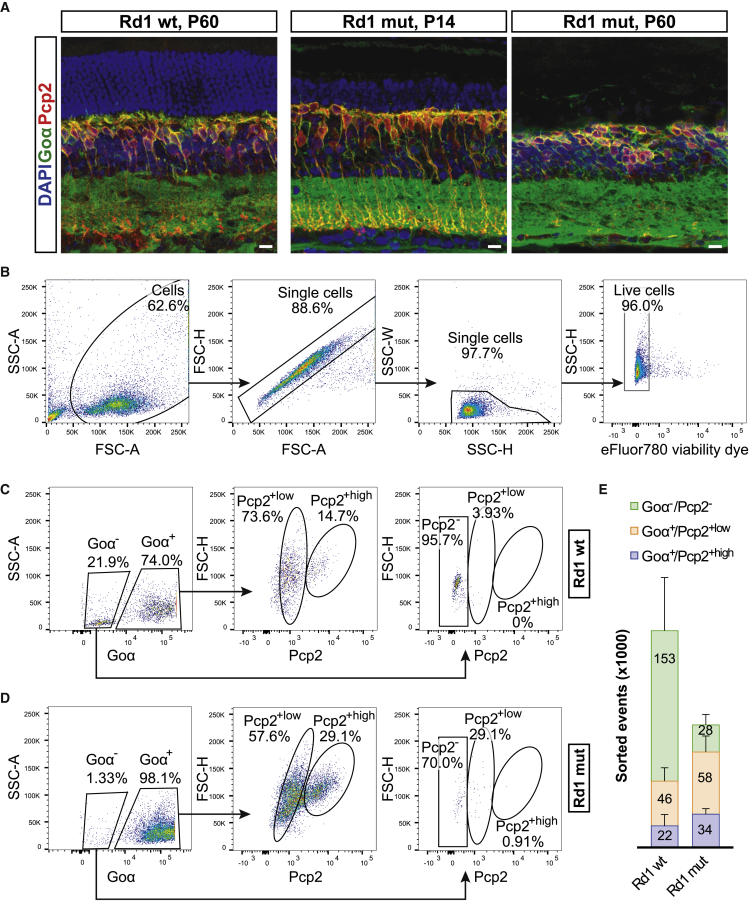
ON bipolar cell isolation from Rd1 WT and mut retinas (A) Confocal microscopy images of IHC-labeled retinal cross-sections showing the expression of ON bipolar cell markers Goα and Pcp2 in healthy Rd1 WT (P60) and degeneration model Rd1 mut at early (P14) and late (P60) stages of degeneration. See also Figure S1 for markers excluded from the sorting panel. Scale bars, 20 μm. (B) Representative FACS dot plots from P60 animals depicting the initial gating strategy for sorting only viable single cells. (C and D) Representative FACS dot plots from P60 Rd1 WT (C) and Rd1 mut (D) retinas showing the sorting gates based on Goα and Pcp2 expression and the representation of the three sorted populations (Goα^−^/Pcp2^−^, Goα^+^/Pcp2^+low^, and Goα^+^/Pcp2^+high^). The preparation of the samples is exemplified in Figure S2. (E) Quantitative representation of the sorted events for each subpopulation obtained from Rd1 WT (N = 6) and Rd1 mut (N = 11) retinas. Error bars shown are SEM.

In order to develop a protocol for sorting ON bipolar cells, cytoplasmic markers were assessed for their specific and stable expression in this cell population. Aiming to develop a method suitable for both healthy and degenerating retinas, we used *Pde6b*^*WT*^ (herein Rd1 wild-type [WT] or WT) and *Pde6b*^*rd1*^ mutant (Rd1 mut or mut) mice, where the latter served as a model of early onset and fast-progressing retinal degeneration^21^, ^22^, ^23^ (Figure S1A). We performed immunohistochemistry (IHC) staining of Rd1 WT and mut retinas to validate whether known markers Pcp2,^24^^,^^25^ Goα,^25^^,^^26^ and Cacna1s^25^^,^^27^ are specifically expressed in ON bipolar cells in healthy and diseased retina. Indeed, a clear cytoplasmic signal for Pcp2 and Goα was detected in the inner nuclear layer (INL) of WT and mut retinas both at the early (postnatal day [P]14) and at later stage (P60) of retinal degeneration, when most photoreceptors have already been lost (Figure 1A). Conversely, Cacna1s protein, which localizes in the dendritic spines of ON bipolar cells in the WT retina, was absent in mut tissue already at the early stage of degeneration (Figure S1B). We therefore selected Pcp2 and Goα for further characterization using fluorescence-activated cell sorting (FACS).

### FACS-based isolation of two retinal subpopulations after co-labeling with Goα and Pcp2

We acutely isolated retinas from 2-month-old Rd1 WT and mut animals and pooled both retinas from each animal before proceeding with papain dissociation, which preserves neuronal viability and has previously been described to allow for good separation of retinal cells.^28^ Subsequently, a standard immunofluorescence procedure was followed to label the retinal suspension with intracellular markers Pcp2 and Goα, which required a fixation and permeabilization step (Figure S2). Dissociated cells were first stained with a viability dye to allow for the discrimination of live and dead cells while sorting, followed by a mild fixation/permeabilization step. After immunolabeling, the retinal cell suspensions were processed via FACS, where baseline gates were first set to discriminate viable single cells from debris and cell aggregates (Figure 1B). A mean viability of nearly 95% of sorted cells was obtained (Figure 1B), indicating that the dissociation process was well tolerated.

Next, we proceeded with the gating strategy for the actual sorting. As observed in the IHC analysis initially performed, Goα showed a leaky expression in the IPL, whereas Pcp2 signal was confined to the cytoplasm of the ON bipolar cells (Figure 1A). Accordingly, viable single cells were first gated with respect to Goα expression and subsequently with respect to Pcp2 (Figures 1C and 1D). In order to characterize the entire Pcp2^+^ population, we identified and sorted two subpopulations, namely Goα^+^/Pcp2^+high^ and Goα^+^/Pcp2^+low^, while as a negative control we collected Goα^−^/Pcp2^−^ cells. Notably, the mean number of sorted events for each population was different between the two mouse lines, with approximately one order of magnitude difference in the Goα^−^/Pcp2^−^ population (Figure 1E), reflecting the loss of photoreceptors in the Rd1 mut retina at the adult (late) stage.

Hence, we concluded that the dissociation allows for high cell viability prior to fixation and that co-labeling with Pcp2 and Goα leads to the discrimination of two subpopulations within the positive cell pool, namely Goα^+^/Pcp2^+high^ and Goα^+^/Pcp2^+low^.

### The Goα^+^/Pcp2^+high^ cell population is exclusive for ON bipolar cells in mice

After sorting the three populations, we processed the cells for RNA isolation to determine the identity of the sorted cells. Given that our protocol required fixation and permeabilization in order to label intracellular proteins, we chose a commercial formalin-fixed and paraffin-embedded (FFPE) kit to revert formaldehyde modifications of nucleic acids and isolate RNA.

**Figure 2:**
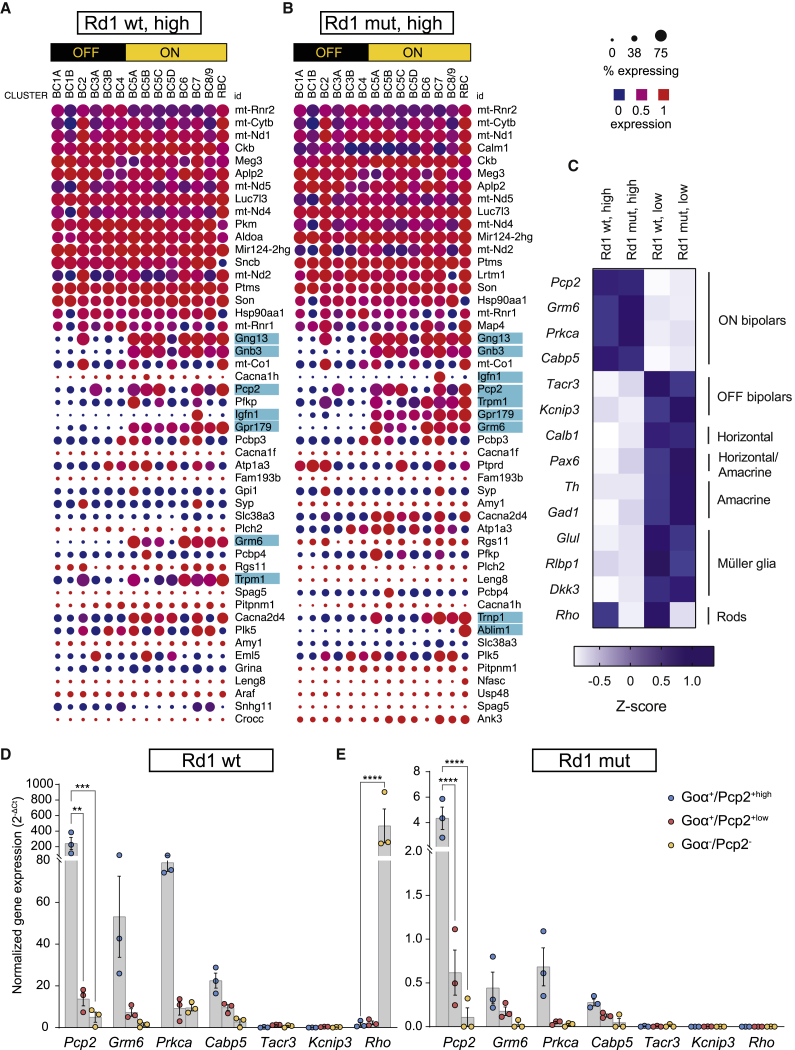
Characterization of sorted populations from Rd1 WT and mut retinas (A and B) Dot plot illustrations of gene expression in bipolar cells from Rd1 WT (A) and mut (B) retinas, after comparative analysis of the top 50 genes from our RNA-seq with publicly available single-cell RNA-seq data (the highest read counts on the Single Cell Portal; see Materials and methods for more details). The data obtained from the high population of both genotypes (see Figure S3 for the low population data) were compared with those from a previous publication,^25^ where a single-cell RNA-seq approach in the retina was used. ON and OFF bipolar subtypes are shown, with the individual bipolar subtype cluster below. Among the 50 submitted genes, only those that found a match are displayed. The size of the circle indicates the percentage of cells expressing a gene in the corresponding single-cell RNA-seq cluster, while the color indicates its mean level of expression. Highlighted in cyan are the genes that show a high expression predominantly if not exclusively in ON bipolar cells. (C–E) *Z* score calculation based on the read counts from the RNA-seq analysis (C), with emphasis on cell-population-specific genes further analyzed in (D) and (E). (D and E) Quantitative reverse-transcriptase PCR (qRT-PCR) of all sorted populations for genes expressed in ON bipolar (*Pcp2*, *Grm6*, *Prkca*, and *Cabp5*), OFF bipolar (*Tacr3* and *Kcnip3*), and photoreceptor cells (*Rho*) in both WT (D) and Rd1 mut (E) mice. N = 3 biological and technical replicates. Two-way ANOVA with Dunnett’s multiple comparisons test. Error bars indicate SEM. Adjusted p values: ∗∗p < 0.01, ∗∗∗∗p < 0.0001.

The extracted RNA (Table 1) was used to obtain gene expression profiles of the sorted populations using RNA sequencing (RNA-seq) analysis of the Goα^+^/Pcp2^+high^ and Goα^+^/Pcp2^+low^ subpopulations (Table S1). The top 50 genes in Goα^+^/Pcp2^+high^ and Goα^+^/Pcp2^+low^ populations were then selected and compared to the gene expression across bipolar cell types in a published single-cell RNA-seq database^25^ (Figures 2A and 2B; Figures S3A and S3B). For both Rd1 WT and mut samples, genes with high expression in ON bipolar cells (including those with specific enrichment in ON versus OFF bipolar cells) were strongly represented in the Goα^+^/Pcp2^+high^ population (cyan highlight; Figures 2A and 2B; Figures S3A and S3B). This result was further confirmed by the enrichment of ON bipolar markers (*Pcp2*, *Grm6*, *Prkca*, and *Cabp5*) and the depletion of OFF bipolar (*Kcnip3* and *Tacr3*), horizontal (*Calb1* and *Pax6*), amacrine (*Pax6*, *Th*, and *Gad1*), Müller glia (*Glul*, *Rlbp1*, and *Dkk3*), and rod photoreceptor (*Rho*) markers from the Goα^+^/Pcp2^+high^ population (Figure 2C). *Rho* was the only exception, as its expression was detected in the Goα^+^/Pcp2^+high^ population of Rd1 WT animals, although at lower levels than in the Goα^+^/Pcp2^+low^ subpopulation (Figure 2C). However, this result could be apportioned to the >1 log unit higher number of rod photoreceptors compared to bipolar cells in the WT mouse retina.^29^

**Table 1:** Total RNA amount extracted from the analyzed samples

Population	RNA (mean ± SD) (ng)		
	Rd1 WT	Rd1 mut	NHP quadrants
Goα^+^, Pcp2^+high^	443 ± 160	270 ± 146	1,100 ± 354
Goα^+^, Pcp2^+low^	421 ± 205	383 ± 261	812 ± 300
Goα^−^, Pcp2^−^	246 ± 88	221 ± 140	961 ± 409
Goα^−^, Pcp2^+low^	N/A	N/A	753 ± 550

N/A, not applicable.

To further validate these findings, we reverse transcribed RNA and analyzed the expression of selected marker genes using quantitative reverse transcriptase PCR (qRT-PCR). With this, we confirmed the enrichment of ON bipolar markers (*Pcp2*, *Grm6*, *Prkca*, and *Cabp5*) in the Goα^+^/Pcp2^+high^ cell population from both Rd1 WT and mut animals, with either no or minimal detection of the OFF bipolar markers *Kcnip3* and *Tacr3*^30^ or the rod photoreceptor marker *Rho* (Figures 2D and 2E). The same ON bipolar markers were also detected in the Goα^+^/Pcp2^+low^ population, although at lower expression levels than in the Goα^+^/Pcp2^+high^ population (Figures 2D and 2E), thus corroborating the distinction of two Pcp2^+^ subpopulations with our sorting protocol. As expected, the rod marker *Rho* was highly enriched in the Goα^−^/Pcp2^−^ control population of Rd1 WT samples (Figure 2D), but absent from corresponding Rd1 mut samples (Figure 2E), well in agreement with the lack of rod photoreceptors in adult (late) stage Rd1 mut mice.

With this, we show that our sorting pipeline is compatible with sensitive detection methods, such as qRT-PCR and RNA-seq analysis, which we used to verify the exclusivity of ON bipolar cells in the Goα^+^/Pcp2^+high^ subpopulation.

### ON bipolar cells from NHP retina are selectively labeled and isolated using Goα and Pcp2

**Figure 3:**
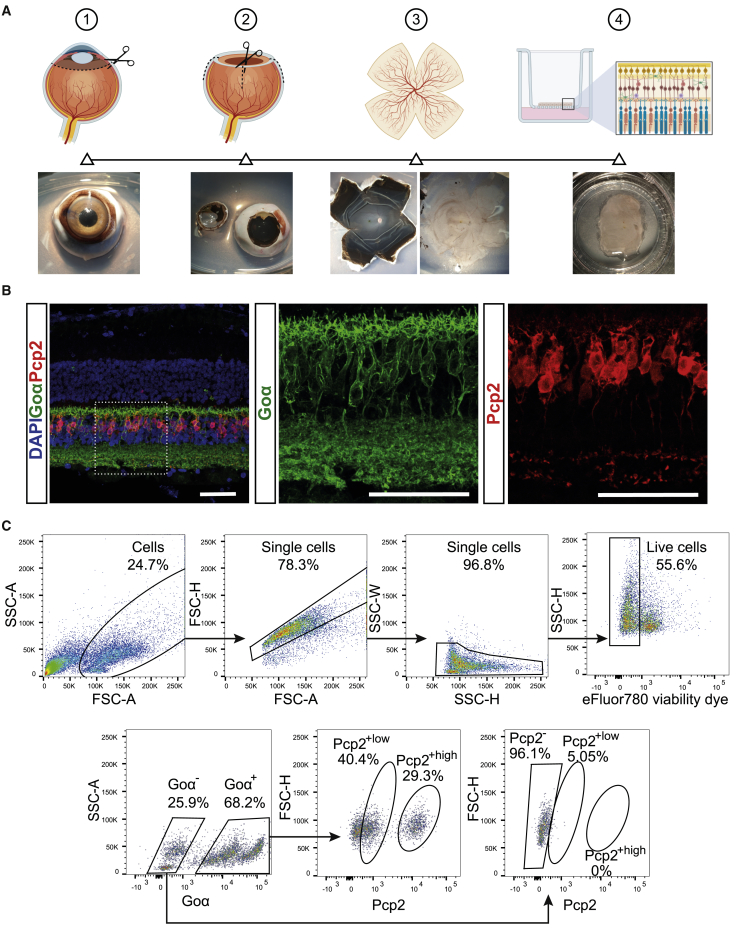
Isolation of ON bipolar cells from NHP retina after overnight culture (A) Graphical representation of the retinal explant preparation accompanied by sample images. (B) Confocal microscopy images of IHC-labeled NHP retina, showing the expression of ON bipolar cell markers Goα and Pcp2, whose localization is better observed in the adjacent magnified panels. Scale bars, 50 μm. (C) Representative FACS dot plots depicting the initial gating strategy for sorting only viable single cells. The preparation of the samples is exemplified in Figure S2. (D) Representative FACS dot plots showing the sorting gates based on Goα and Pcp2 expression and the representation of the four sorted populations (Goα^−^/Pcp2^−^, Goα^+^/Pcp2^+low^, Goα^−^/Pcp2^+low^ and Goα^+^/Pcp2^+high^).

Given the high efficiency and specificity of the established ON bipolar isolation pipeline in mice, we wanted to transpose the approach on the NHP retina, as it better approximates the human physiology. To this end, we extracted the retinas from naive NHP eyes and carefully dissected them into quadrants (Figure 3A). For logistical reasons and to allow for *ex vivo* manipulation, we chose to maintain the tissue overnight as retinal explant cultures with the outer nuclear layer (ONL) facing down on hydrophilic methylcellulose culture inserts (Figure 3A). Subsequent IHC analysis showed that Goα and Pcp2 antibodies efficiently labeled a subpopulation within the INL, which could be inferred as the ON bipolar cell population by their intra-layer localization and distribution (Figure 3B). After the overnight culture, the NHP retinal explant tissue was harvested and dissociated either as a whole or as small 6-mm-diameter biopsy punches. The papain-based dissociation was adapted with respect to reagent quantities to compensate for the larger tissue amount, thickness and composition. Contrary to the mouse samples, we obtained a lower cell viability from the NHP samples (almost 56%; Figure 3C), which could be due to the lengthier process of retinal isolation from the NHP eye as well as the overnight culture step. Nevertheless, the viable cell fraction had a distinct Goα^+^ population, which was further discriminated into Pcp2^+high^ and Pcp2^+low^ subpopulations. Interestingly, the Goα^−^ population was also divided into Goα^−^/Pcp2^+low^ and Goα^−^/Pcp2^-^, although the former accounted for only a small fraction of the entire Goα^−^ population (Figure 3D).

Overall, the same dissociation procedure and antibody panel used for mouse samples could be adapted for NHP samples and resulted in two distinct Pcp2^+^ subpopulations within the Goα^+^ pool of the NHP retina, similar to the Rd1 WT and mut retinas.

### The Goα^+^/Pcp2^+high^ subpopulation from NHP retina is predominantly ON bipolar cells

**Figure 4:**
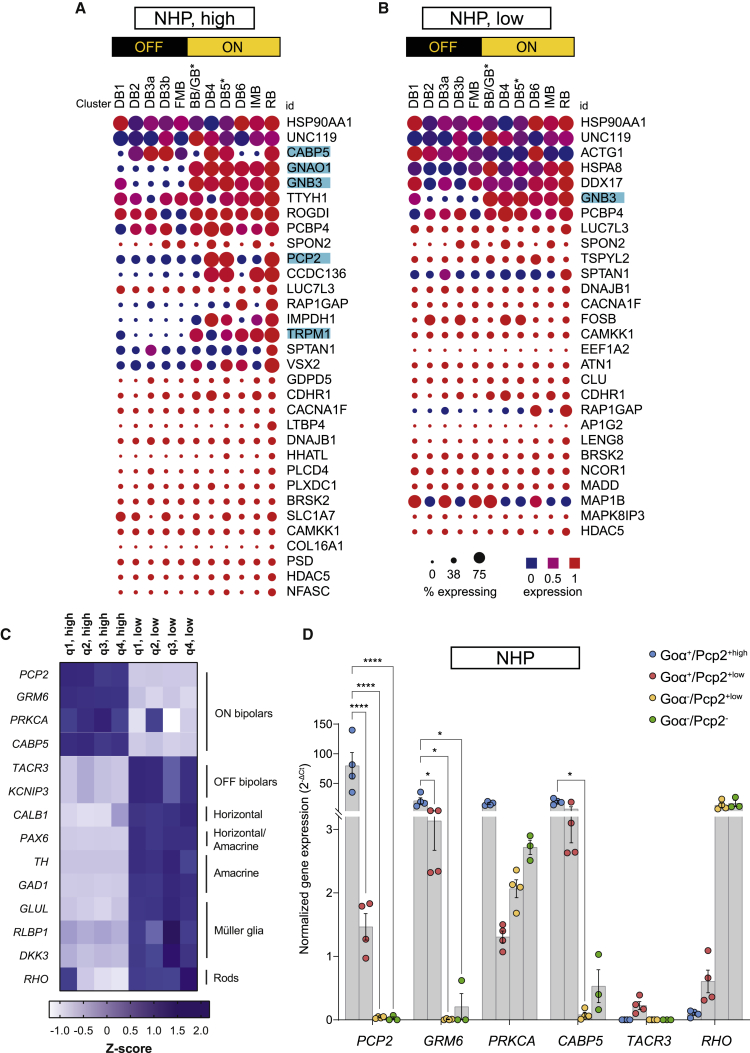
Characterization of sorted population from NHP retinas (A and B) Dot plot illustration of gene expression in bipolar cells from the high (A) and low (B) populations after comparative analysis of the top 50 genes from our RNA-seq analysis with publicly available single-cell RNA-seq data (the highest read counts on the Single Cell Portal, see Materials and methods for more details). The mean of the read count values from each quadrant was used to select the top genes. The data were compared with those from a previous publication,^31^ where a single-cell RNA-seq approach in the retina was used. ON and OFF bipolar subtypes are shown, with the individual bipolar subtype cluster below. Among the 50 submitted genes, only those that found a match are displayed. The size of the circle indicates the percentage of cells expressing a gene in the corresponding single-cell RNA-seq cluster, while the color indicates its mean level of expression. Highlighted in cyan are the genes that show a high expression predominantly if not exclusively in ON bipolar cells. (C) *Z* score calculation based on the read counts from the RNA-seq analysis, with emphasis on cell population-specific genes. (D) qRT-PCR of all sorted populations for genes expressed in different cell populations. A total of four different quadrants from the same retina were processed and three technical replicates were performed. Two-way ANOVA with Dunnett’s multiple comparisons test. Error bars indicate SEM. Adjusted p values: ∗p < 0.05, ∗∗∗∗p < 0.0001.

To characterize the Goα^+^/Pcp2^+high^ subpopulation sorted from the NHP retina and to investigate whether it is, as in mice, enriched in ON bipolar cells, we isolated RNA (Table 1), performed RNA-seq analysis of the Goα^+^/Pcp2^+high^ and Goα^+^/Pcp2^+low^ subpopulations (Table S1), and then used the top 50 genes with the highest reads to find matches in the single-cell data made available by the Sanes lab^31^ (Figures 4A and 4B). Among those genes, several of the Goα^+^/Pcp2^+high^ subpopulation were known markers of ON bipolar cells (cyan highlight; Figure 4A), while only *GNB3* was found in the Goα^+^/Pcp2^+high^ and Goα^+^/Pcp2^+low^ subpopulations (cyan highlight; Figure 4B). Indeed, previous data^31^ have reported *GNB3* expression also in the OFF bipolar subtype DB1, potentially explaining the detection of this gene in both sorted subpopulations. The same markers analyzed for the mouse showed high expression levels in the NHP Goα^+^/Pcp2^+high^ subpopulation, further indicating an enrichment of ON bipolar cells (Figure 4C). Specifically, *PCP2*, *GRM6*, *PRKCA*, and *CABP5* showed an almost exclusive expression in the Goα^+^/Pcp2^+high^ subpopulation, while non-ON bipolar markers such as *KCNIP3*, *TACR3*, *CALB1*, *PAX6*, *TH*, *GAD1*, *GLUL*, *RLBP1*, *DKK3*, and *RHO* had the highest reads in the Goα^+^/Pcp2^+low^ subpopulation for all processed quadrants (Figure 4C). Only one quadrant had detectable *RHO* expression in the Goα^+^/Pcp2^+high^ subpopulation, possibly as a result of photoreceptor contamination in this specific sample.

To further validate this RNA-seq data, we reverse transcribed RNA from the four sorted cell populations, namely Goα^+^/Pcp2^+high^, Goα^+^/Pcp2^+low^, Goα^−^/Pcp2^+low^, and Goα^−^/Pcp2^−^, and analyzed the expression of selected marker genes using qRT-PCR. As observed in mice, the Goα^+^/Pcp2^+high^ subpopulation displayed the highest levels of the ON bipolar markers *PCP2*, *GRM6*, *PRKCA*, and *CABP5*, whose expression was instead reduced in the Goα^+^/Pcp2^+low^ subpopulation as well as in the Goα^−^ pool (Figure 4C). Strikingly, little to no expression could be detected for the OFF bipolar marker *TACR3* and the rod marker *RHO* in the Goα^+^/Pcp2^+high^ fraction, thereby sustaining the high bias of this sorting approach toward a pure ON bipolar cell population. Conversely, a detectable *TACR3* expression appeared in the Goα^+^/Pcp2^+low^ subpopulation, as already observed in the same population of mouse specimens (Figure 2).

Overall, we could show that the Goα^+^/Pcp2^+high^ subpopulation isolated from NHP retinas contains ON bipolar cells with a high degree of purity and that downstream molecular analyses can be efficiently performed regardless of the extensive sample manipulation.

### Viral genomes are detected and retrieved from sorted cells after AAV subretinal delivery

After assessing the impact of fixation on downstream analyses focusing on RNA, we set out to perform a proof-of-concept study to demonstrate that sorted Goα^+^/Pcp2^+high^, Goα^+^/Pcp2^+low^ and Goα^−^/Pcp2^−^ populations could be used for screening viral vector libraries. For this purpose, Rd1 mut mice and NHPs were subretinally injected with 1E9 viral genomes of AAV libraries,^32^^,^^33^ whose genome contained the AAV *rep* and modified *cap* genes. Retinas were harvested after an in-life period of 24 h and 1 week for mice and NHPs, respectively. Subsequently, all retina samples were processed for sorting with the previously established procedure, followed by isolation of genomic DNA (gDNA) using the FFPE kit (Table 2). The Droplet Digital PCR (ddPCR)-based analysis confirmed the presence of the viral gene *rep2*, thus verifying the presence of AAV library genomes in the isolated cell populations. Interestingly, although the total gDNA yield was comparable between sorted populations originating from mouse and NHP retinas, the number of total viral genomes detected in NHP samples was two orders of magnitude greater, specifically in the Goα^−^/Pcp2^−^ population (Table 2). The quantity and quality of the detected viral genomes were deemed sufficient for the production of AAV sublibraries, both from mouse and NHP sorted populations, thus demonstrating the applicability of this sorting protocol as part of a wider vector or library screening pipeline.

**Table 2:** Total gDNA extracted and total AAV viral genomes detected in analyzed samples

Population	gDNA (mean ± SD) (ng)		Viral genomes (vg) (mean ± SD)	
	Mouse	NHP	Mouse	NHP
Goα^+^, Pcp2^+high^	203 ± 129	273 ± 172	7,271 ± 5,092	11.916 ± 7,968
Goα^+^, Pcp2^+low^	234 ± 230	236 ± 178	22,397 ± 29,370	90,804 ± 93.082
Goα^−^, Pcp2^−^	214 ± 204	408 ± 118	22,746 ± 17,717	2.625.000 ± 1,965,003

## Discussion

Neurodegeneration, whether in the brain or retina, is not innately reversible in mammals. Irrespective of the underlying cause, depleted photoreceptors in the retina are not regenerated in mammals, thus leading to various forms of blindness. However, the retinal network that remains structurally intact in the absence of photoreceptors could be repurposed to generate light-induced responses. An example of this approach is the use of optogenetics, a strategy where a gene expressing a light-sensing protein is introduced into the remaining neurons to render them photosensitive. Given their position and function within the ON circuit, ON bipolar cells are an ideal recipient of optogenetic tools. However, selective and efficient viral or non-viral vectors that can deliver these tools to ON bipolar cells, both in animal models and humans, are still missing. In order to facilitate the development of such modalities, we have established a method that exclusively isolates ON bipolar cells in at least two different species (mice and NHPs) in healthy and degenerated retinas.

Purification of ON bipolar cells has so far depended on transgenic mouse lines.^25^ This restriction to rodents limits the translatability of the results, as murine and human retinas differ substantially. Larger mammals, such as dogs and NHPs, are more relevant to humans, but no transgenic lines or other strategies exist to isolate ON bipolar cells from these species. This limitation holds true for other cell types in the retina as well, leading to the establishment of alternative cell isolation strategies.^34^ To address this unmet need, we developed a method that is applicable to a broad range of *in vivo* models, in particular primate models.

The absence of an isolation method for ON bipolar cells is largely due to the lack of a specific cell surface marker or reliable antibodies against known membrane proteins (e.g., mGluR6). In turn, this prevents the use of methods that strictly rely on the recognition of surface antigens, e.g., magnetic-activated cell sorting. To overcome this limitation, we opted for an approach that combines FACS with intracellular markers. Fixed retinas were stained for the intracellular ON bipolar markers Pcp2 and Goα, and then sorted based on their antibody expression profile. These markers were selected for their specificity and consistency in both healthy and degenerating retinas. The gating strategy to determine the Pcp2^+^ population within the Goα^+^ population was based on the antibody profile obtained via IHC, where Goα stained the IPL in addition to ON bipolar cells (Figure 1). A widespread signal for Pcp2 was detected, which led us to hypothesize the existence of two subpopulations, namely Pcp2^+high^ and Pcp2^+low^. This distinction could indicate different levels of protein expression, as qualitatively suggested by IHC, and may also reflect more subtle differences within the ON bipolar cell pool that are not yet investigated. This hypothesis is supported by the results of the single-cell RNA-seq analysis performed by the Sanes group,^25^ which show a variable *Pcp2* expression across ON bipolar cells as well as a detectable expression in at least one OFF bipolar subpopulation.

For subsequent characterization following sorting, we extracted RNA and gDNA from Goα^+^/Pcp2^+high^, Goα^+^/Pcp2^+low^, and Goα^−^/Pcp2^−^ populations after reversing the mild fixation used during immunolabeling. One potential caveat of fixing the cells is that the quality of the RNA and DNA material could be compromised by the treatment, even after reverting the cross-links by Proteinase K treatment. We therefore assessed the impact of fixation on sensitive downstream analyses. To perform reverse transcription, we used two different commercial kits that proved to be interchangeable in terms of qRT-PCR results. More importantly, RNA-seq of sorted populations was successfully performed with samples from both mice and NHPs, with results that matched the qRT-PCR data; this eased any concern about detrimental damage of RNA after fixation. Of note, by comparing our sequencing data to publicly available single-cell RNA-seq data we not only confirmed the identity of the isolated cell populations, but also showed that the two approaches result in a similar transcriptional signature of ON bipolar cells. Although single-cell techniques enable the simultaneous analysis of distinct cell populations and provide a detailed view of cell-specific gene expression patterns, our method proves to be an amenable and, due to the use of less expensive bulk RNA-seq technology, cost-efficient alternative that can be potentially adapted to isolate other cell types.

The quality of the isolated DNA from sorted populations was also tested, with the purpose of detecting viral genomes during high-throughput library screens that target ON bipolar cells. To this end, we administered AAV libraries in mice and NHPs prior to retinal tissue harvest and then successfully recovered viral DNA from ON bipolar cells in quantities suitable for sublibrary production. While it is possible to reverse the cross-linking of fixation in order to isolate nucleic acids and recover viral DNA, we cannot exclude that this process may not be equally compatible with other downstream applications. Therefore, the identification of a specific surface marker for live ON bipolar isolation across species remains of interest.

The consistent profile of the ON bipolar markers Goα and Pcp2 across disease states and species was crucial for the translatability of this method. An alternative combination with a generic bipolar marker that does not discriminate between ON and OFF bipolar cells (e.g., Chx10^35^, ^36^, ^37^) together with Pcp2 could have been used, following the general-to-specific approach often suggested for sorting strategies. However, the differential expression of such a generic marker in bipolar cells across species could have interfered with the universal applicability of this method. As evidenced by the sorts of WT samples from mice (Figure 2) and NHPs (Figure 4), the purity of a sorted population can be compromised even when cell-specific markers are used. Indeed, *RHO* transcripts were detected in some Goα^+^/Pcp2^+high^ and most Goα^+^/Pcp2^+low^ subpopulations on account of the much higher number of photoreceptors present in the retina compared to any other cell type,^29^ further underlining the importance of the sorting panel.

Acute cell isolation using tissue dissociation followed by cell sorting can result in severely compromised neuronal viability, given the overall mechanical strain and duration of the procedure. While the fixation step between dissociation and sorting allowed us to preserve a high viability (96%) for both mouse samples, NHP cells did not survive equally well, which could be apportioned to the time between necropsy and retina isolation from the eye (approximately 6 hours), the sample handling, or the overnight retinal culture before sorting (Figure 3A). The latter could be avoided by minimizing the time between necropsy and tissue isolation, and by processing the retinas without interruptions. Alternatively, immediate fixation of the dissociated cells and subsequent sorting 24 h later can minimize cell death, without affecting the sorting outcome (data not shown). Therefore, the time between animal necropsy and fixation of dissociated cells seems to be crucial for maintaining cell viability.

While we provide evidence that our sorting method can successfully isolate ON bipolar cells from mouse and NHP retina, its validation using human retinal tissue remains a future task. Nevertheless, as drug-delivery tools such as viral vectors and nanoparticles^38^ are screened using animal models, particularly rodents and NHPs in the case of ocular diseases, our procedure to exclusively sort ON bipolar cells from these species without substantial adaptations is highly relevant. We therefore envision that this method could facilitate the development of next-generation, cell type-specific gene delivery or genetic engineering tools to treat late-stage retinal degeneration and help advance our understanding of ON bipolar cell biology across species and disease states.

## Materials and methods

### Animals

Experiments were mainly conducted on *Pde6b*^*WT*^ (Rd1 WT) and *Pde6b*^*rd1*^ (Rd1 mut) mice (*Mus musculus*) at 2–3 months of age, unless differently specified in the text, and with no specific sex bias. All mice were chow fed and provided with abundance of water, while being kept in a 12-h light/12-h dark cycle. All animal protocols were performed with permission of local authorities (District Government of Upper Bavaria) and in accordance with the German laws on animal welfare (Tierschutzgesetz).

Cynomolgus monkeys (*Macaca fascicularis*) were treated and taken care of at the Covance Preclinical Services test facility in Münster, Germany. The study was approved by the local Institutional Ethics Board and conducted under non-good laboratory practice (GLP) conditions. The age of animals ranged between 2 and 5 years. The weight of animals was between 3.0 and 15.0 kg in males and 2.0 and 6.0 kg in females before treatment. An animal health assessment was performed by a qualified veterinarian to confirm the suitability of each animal for the study.

### Subretinal injections

In mice, subretinal injections were done with slight modifications to our standard protocol.^39^^,^^40^ In brief, mice were anesthetized by intraperitoneal injections of ketamine (0.1 mg/g) and xylazine (0.02 mg/g). Tropicamide eye drops were applied to dilate the pupils (Mydriaticum, Pharma Stulln). Injections were performed with the UMP3T-1 microinjection syringe pump using the NanoFil sub-microliter injection system (World Precision Instruments) equipped with a 34G beveled needle under an OPMI 1 FR pro surgical microscope (Carl Zeiss). For subretinal injections, the needle bevel was turned up while penetrating the sclera, choroid, and retina. Subretinal positioning of the needle was confirmed using a surgical stereomicroscope. One microliter containing 1E9 total viral genomes of AAV library solution, prepared as previously described,^32^^,^^33^ was injected into the subretinal space and the needle was held for >20 s before retracting to avoid backflow.

For NHP surgeries, performed under general anesthesia (isoflurane) in operating theaters, animals were placed in dorsal recumbence and their eyes were dilated with topical tropicamide (1%). The orbital region of the operating eye was treated with 10% povidone iodine solution followed by flushing of conjunctival fornixes with 0.5% of the solution. Sterile surgical drape was applied, pediatric speculum inserted, and a temporal canthotomy performed for improved ocular surgical access. 23G three-point pars plana core and peripheral vitrectomy was performed under the operating microscope as per human surgeries. The subretinal viral library injection was performed by a two-step procedure as in human gene therapy trials. First, a localized retinal detachment was induced at the macula with basic salt solution (Alcon Laboratories) using a 41G cannula (Dutch Ophthalmic Research Center [DORC] 1270.EXT). Second, AAV library solution (up to 200 μL) was injected slowly via the same retinotomy site into the preformed bleb using a foot pedal-controlled injection system (PentaSys II; Ruck). At the end of the surgery, subconjunctival cefuroxime (125 mg) and dexamethasone (2 mg) were administered to the operated eye. Postoperatively, animals were treated with topical moxifloxacin (0.5%) and prednisolone (1%) eye drops for 1 week. All animals were systemically immunosuppressed with intramuscular prednisone (1 mg/kg) from day −2 to day 5 after the procedure.

### Retinal explant culture

Eyes from NHPs were transferred into CO_2_-independent medium (#18045-054, Thermo Fisher Scientific) after surgical removal. Upon arrival (4–5 h after surgery), the eyes were sectioned to expose the posterior eye cup, which was subsequently cut into quadrants. The retina was then gently peeled off the underlying retinal pigmented epithelium and further sectioned to separate four peripheral quadrants, which were later transferred to hydrophilic PTFE cell culture inserts with 30-mm diameter and 0.4-μm pore size (#PICMORG50, Millipore) with the ONL of the retina placed facing the membrane. Retinal explants were cultured in Neurobasal-A medium (#10888022, Thermo Fisher Scientific) supplemented with 2 mM l-glutamine (#25030024, Thermo Fisher Scientific), B27 supplement (#17504044, Thermo Fisher Scientific), and antibiotic-antimycotic (#15240062, Thermo Fisher Scientific). Retinal explants were kept overnight at 37°C, 5% CO_2_ and processed for cell sorting the next day.

### Dissociation of retinas

Acutely isolated mouse retinas were pooled from one animal for dissociation. The Papain Dissociation System (Worthington Biochemical) was used as previously described.^28^ Briefly, retinas were minced into smaller pieces and incubated in a mixture of papain and DNase at 37°C for 45–60 min, in the presence of mild agitation (600 rpm). A solution containing DNase/BSA/ovomucoid in Earle’s balanced salt solution (EBSS) was then added, followed by BSA/ovomucoid and more pipetting, until no more retinal pieces were visible. The cell suspension was then centrifuged at 0.4 relative centrifugal force (rcf) for 5 min at 4°C and the supernatant discarded.

For NHP retinas, after the night in culture the explant quadrants were processed as a whole or punctured with a 6-mm biopsy punch (#270038, Stiefel Einweg biopsy punch, Stiefel Laboratorium) to excise a sample for subsequent dissociation. The same protocol followed for mouse retinas was used, with the only difference being the upscaled volumes of the reagents.

### Immunolabeling for cell sorting

All incubation steps were performed on ice and all centrifugation steps were done at 0.4 rcf for 5 min at 4°C. The reagent dilutions were prepared fresh before use and all antibodies were diluted 1:250. The cell pellet obtained from the dissociation was first stained with eBioscience fixable viability dye eFluor 780 (#65-0865-14, Thermo Fisher Scientific) 1:1,000 in PBS for 20 min. The suspension was then centrifuged and the supernatant discarded. Cells were fixed in BD Phosflow fix buffer I (#557870, BD Biosciences) for 20 min, followed by centrifugation and supernatant removal. The cell pellet was then resuspended in BD Phosflow Perm/Wash buffer I (#557885, BD Biosciences) according to the manufacturer’s indications. 10-μl-aliquots of the cell suspension were used as controls for the viability dye and the secondary antibody as well as for a fluorescence-minus-one (FMO) control. The remaining cell suspension was incubated with the primary antibody for Goα (#MAB3073, Merck Millipore) for 20 min. After spinning down to remove the solution and washing once with BD Phosflow Perm/Wash buffer I, the conjugated antibody for Pcp2-Alexa Fluor 647 (#sc-137064 AF647, Santa Cruz) and the secondary antibody Alexa Fluor 488 (#A-21121, Thermo Fisher Scientific) were added to the cells and incubated for 20 min. The suspension was ready for cell sorting after a last centrifugeation and wash step.

### Cell sorting

Cell sorting was performed on a FACSAria Fusion (Becton Dickinson) on BD FACSDiva software v8.0 using a 100-μm nozzle at 20 psi. To this end, cells were filtered through a 40-μm PluriStrainer mini (#43-10040-60, PluriSelect) right before sorting. For downstream RNA/gDNA extraction, single, eFluor 780 negative cells were sorted into PBS, according to their expression levels of Pcp2 and Goα as indicated. Data were analyzed with FlowJo 10.6.1 for Mac OS X. FMO controls or controls without primary antibody were used as gating controls.

### IHC

Coronal cryosections (16–20 μm) were collected serially in a cryostat and incubated at room temperature for 60 min with a blocking solution consisting of 2% BSA, 0.3% Triton X-100 in PBS. The following primary antibodies were diluted in the same solution and incubated overnight at 4°C: mouse anti-Goα (1:500; #MAB3073, Merck Millipore), mouse anti-Pcp2 (1:500; #sc-137064, Santa Cruz), mouse anti-Cacna1s (1:250; #MAB427, Merck Millipore), rabbit anti-recoverin (1:500; #AB5585, Merck Millipore), rabbit anti-iba1 (1:500; #234 013, Synaptic Systems), and rat anti-Cd11b (1:500; #101202, BioLegend). Following several washes in PBS, the sections were incubated for 2 h at room temperature with the following secondary antibodies, all diluted 1:500 in blocking solution: anti-mouse Alexa Fluor 488 (#A-21121), anti-rat Alexa Fluor 488 (#A-21208), anti-rabbit Alexa Fluor 488 (#A-11008), anti-mouse Alexa Fluor 555 (#A-21127), and anti-rabbit Alexa Fluor 647 (#A-32733, all antibodies from Thermo Fisher Scientific). The sections were then washed in PBS and counterstained with DAPI (#D1306, Thermo Fisher Scientific) prior to mounting (#18606-5, Aqua-Poly/Mount).

### Image acquisition and visualization

Images were acquired with an inverted Leica DMI 8 confocal microscope equipped with lasers emitting at the wavelengths 405, 488, 552, and 638 nm, using an objective magnification of ×40 or ×63. The original images, consisting of multiple z stacks, were further processed with the open-source software Fiji^41^ prior to final elaboration in Inkscape. Graphs were made with GraphPad Prism 9.

### Gene expression analysis

RNA and gDNA were isolated simultaneously from the sorted cell populations using the AllPrep DNA/RNA FFPE kit (#80234, QIAGEN) according to the manufacturer’s instructions, and their amount was evaluated with a NanoDrop 2000 spectrophotometer (Thermo Fisher Scientific). Fifty nanograms of RNA was then reverse-transcribed to cDNA using either the SuperScript IV reverse transcriptase (#18090050, Thermo Fisher Scientific) or the RevertAid first-strand cDNA synthesis kit (#K1621, Thermo Fisher Scientific).

For qRT-PCR we designed gene-specific primers, distinct for mouse (see Table S2) and NHP (see Table S3) samples, with an annealing temperature of the primer pairs at 60°C. The PowerUp SYBR Green master mix (#A25742, Thermo Fisher Scientific) was used as a detection method for the QuantStudio 5 real-time PCR platform (Thermo Fisher Scientific).

### RNA-seq

Total RNA was submitted to the Genomic Technologies Core Facility (GTCF) in the Faculty of Biology, Medicine and Health at the University of Manchester. Quality and integrity of the RNA samples were assessed using a 2200 TapeStation (Agilent Technologies) and then libraries generated using the TruSeq stranded mRNA assay (Illumina) according to the manufacturers’ protocols. Briefly, the total RNA was used as input material from which polyadenylated mRNA was purified using poly(T), oligonucleotide-attached magnetic beads. The mRNA was then fragmented using divalent cations under elevated temperature and next reverse-transcribed into first-strand cDNA using random primers. Second-strand cDNA was then synthesized using DNA polymerase I and RNase H. Following a single “A” base addition, adapters were ligated to the cDNA fragments, and the products then purified and enriched by PCR to create the final cDNA library. Adaptor indices were used to multiplex libraries, which were pooled prior to cluster generation using a cBot instrument. The loaded flow cell was then paired-end sequenced (76 + 76 cycles, plus indices) on an Illumina HiSeq 4000 instrument. Finally, the output data were demultiplexed (allowing one mismatch) and BCL-to-Fastq conversion was performed using Illumina’s bcl2fastq software, version 2.20.0.422.

The data have been deposited in ArrayExpress: E-MTAB-9685.

### Visualization of RNA-seq data

Data were sorted according to the number of reads, the first 50 genes in each sorted population were used as a query for the Single Cell Portal of the Broad Institute (for mice, https://singlecell.broadinstitute.org/single_cell/study/SCP3/retinal-bipolar-neuron-drop-seq; for NHP, https://singlecell.broadinstitute.org/single_cell/study/SCP212/molecular-specification-of-retinal-cell-types-underlying-central-and-peripheral-vision-in-primates), and the resulting heatmap was sorted in a descending manner. Only the genes that found a match were also visualized, and therefore for some queries a number <50 is shown in the figures. For NHP data, the database specific for peripheral bipolar cells was selected prior to the analysis.

### Statistical analysis

For statistical analysis of RT-PCR data, GraphPad Prism 9 was used. Three technical replicates per mouse were averaged, and samples from individual mice were treated as one “n.” The mean expression values of the single genes were compared across the different sorted populations by two-way ANOVA, keeping the Goα^+^/Pcp2^+high^ population as the standard for all comparisons. Dunnett’s post hoc test was implemented to compensate for multiple comparisons, and the adjusted p values were therefore used for statistical significance. The significance level of α = 0.05 was accepted (∗p < 0.05, ∗∗p < 0.01, ∗∗∗p < 0.001). Only significant results are shown in the plots.

### Detection of AAV viral genomes

AAV peptide display libraries were prepared as previously described^32^^,^^33^ and quantified using a ddPCR system using *rep2* as the target. To quantify viral concentration of the libraries and therefore the cellular uptake of AAVs from the isolated cells, DNA extracts were quantified using a ddPCR. Virus genome copy numbers were quantified using a TaqMan probe against the AAV2 *rep* gene (forward, 5′-AAGTCCTCGGCCCAGATAGAC-3′; reverse, 5′-CAATCACGGCGCACATGT-3′; TaqMan probe, 5′-FAM-TATCGTCACCTCCAACA-BHQ1-3′), and the mouse and NHP genome copies were quantified using a commercially available TaqMan probe (ddPCR gene expression assay: Rpp30, mouse, catalog no. 10031255, Bio-Rad) for mouse samples and one designed against albumin for NHPs (forward, 5′-ATCTCTCCCTGGCATTGTTG-3′; reverse, 5′-ATCCAAACTCATGGGAGCTG-3′; TaqMan probe, 5′-HEX-TTGCAGATGTCAATGAAAGAGAACCGG-BHQ1-3′). Reactions were processed according to the manufacturer’s instructions and were read in a QX200 ddPCR system (Bio-Rad).

## Supplemental information

Document S1. Figures S1–S3 and Tables S2 and S3

Table S1: RNA-seq data of mouse and NHP samples

Document S2. Article plus supplemental information
