# Supplementary material for: A universal protocol for isolating retinal ON bipolar cells across species via fluorescence-activated cell sorting
Source: Mol Ther Methods Clin Dev. 2021 Jan 26;20:587–600. doi: 10.1016/j.omtm.2021.01.011 (PMC7895692; doi:10.1016/j.omtm.2021.01.011)
Supplement: Document S1. Figures S1–S3 and Tables S2 and S3 [file mmc1.pdf]

OMTM, Volume *20*

Supplemental Information

A universal protocol for isolating retinal ON bipolar cells across species via fluorescence-activated cell sorting

Elisa Murenu, Marina Pavlou, Lisa Richter, Kleopatra Rapti, Sabrina Just, Jasmina Cehajic-Kapetanovic, Neda Tafrishi, Andrew Hayes, Rachel Scholey, Robert Lucas, Hildegard Büning, Dirk Grimm, and Stylianos Michalakis

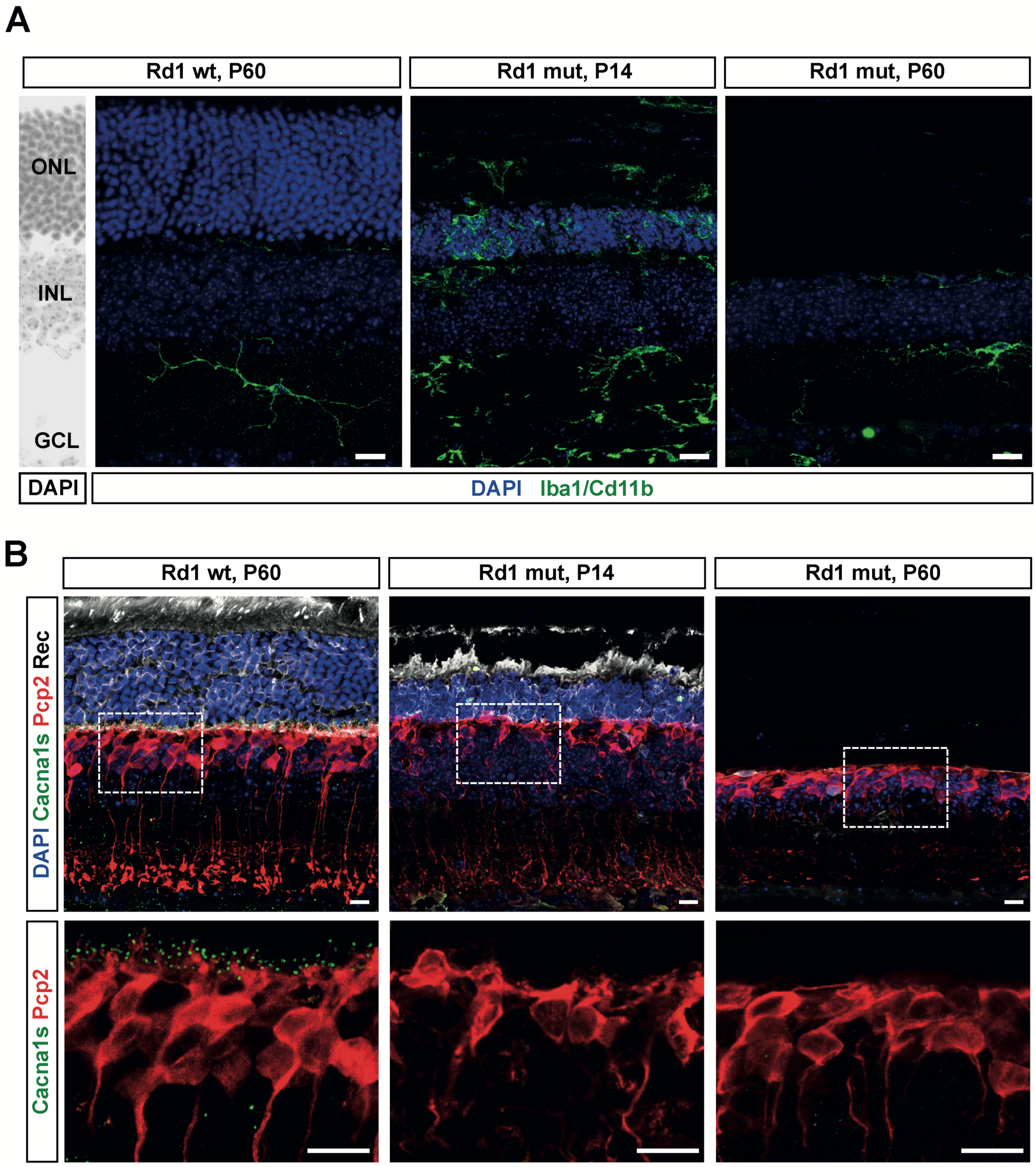

Figure S1. IHC profile of healthy and degenerating mouse retinas.
(A-B) Confocal microscopy images of IHC-labeled retinal slices, in healthy Rd1 wt (P60) and degeneration model Rd1 mut at early (P14) and late (P60) stages of degeneration. (A) The expression of microglia marker Iba-1 coincides with the acute degeneration of photoreceptors in the ONL. (B) ON bipolar markers Cacna1s and Pcp2 are shown together with the photoreceptor marker Recoverin (Rec). The marked rectangle in the upper panel prompts to the magnified images in the lower panel, where the absence of Cacna1s in the Rd1 mut retina is shown, irrespective of degeneration stage. ONL, outer nuclear layer; INL, inner nuclear layer; GCL, ganglion cell layer. Scale bar: 20 µm

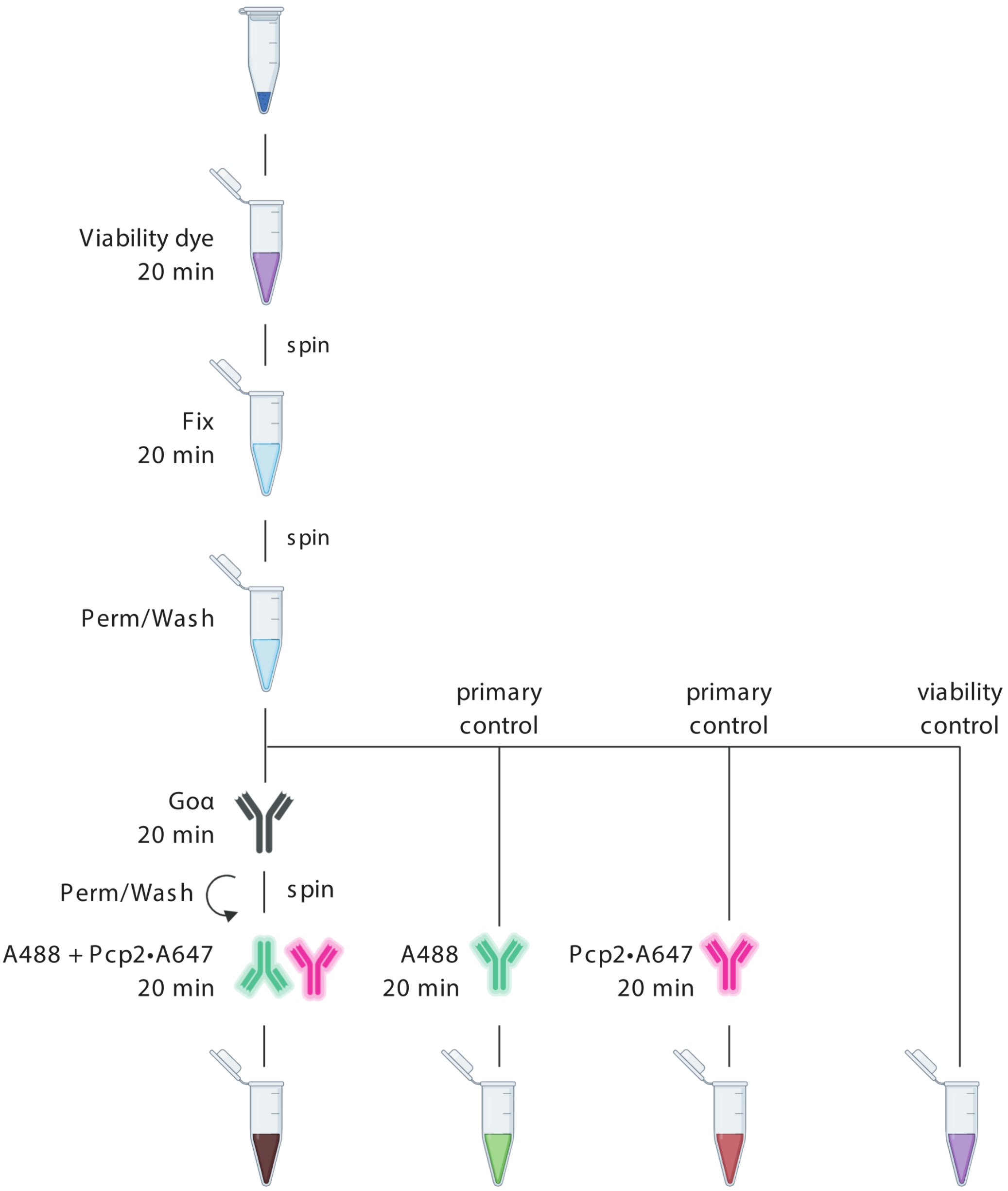

Figure S2. Graphical representation of staining procedure before FACS.
Individual steps of the staining protocol shown schematically in order to ease the reproduction of the procedure and preparation of the appropriate controls (aliquot control I-II and viability control) required during FACS sorting. This schematic was generated using biorender.com.

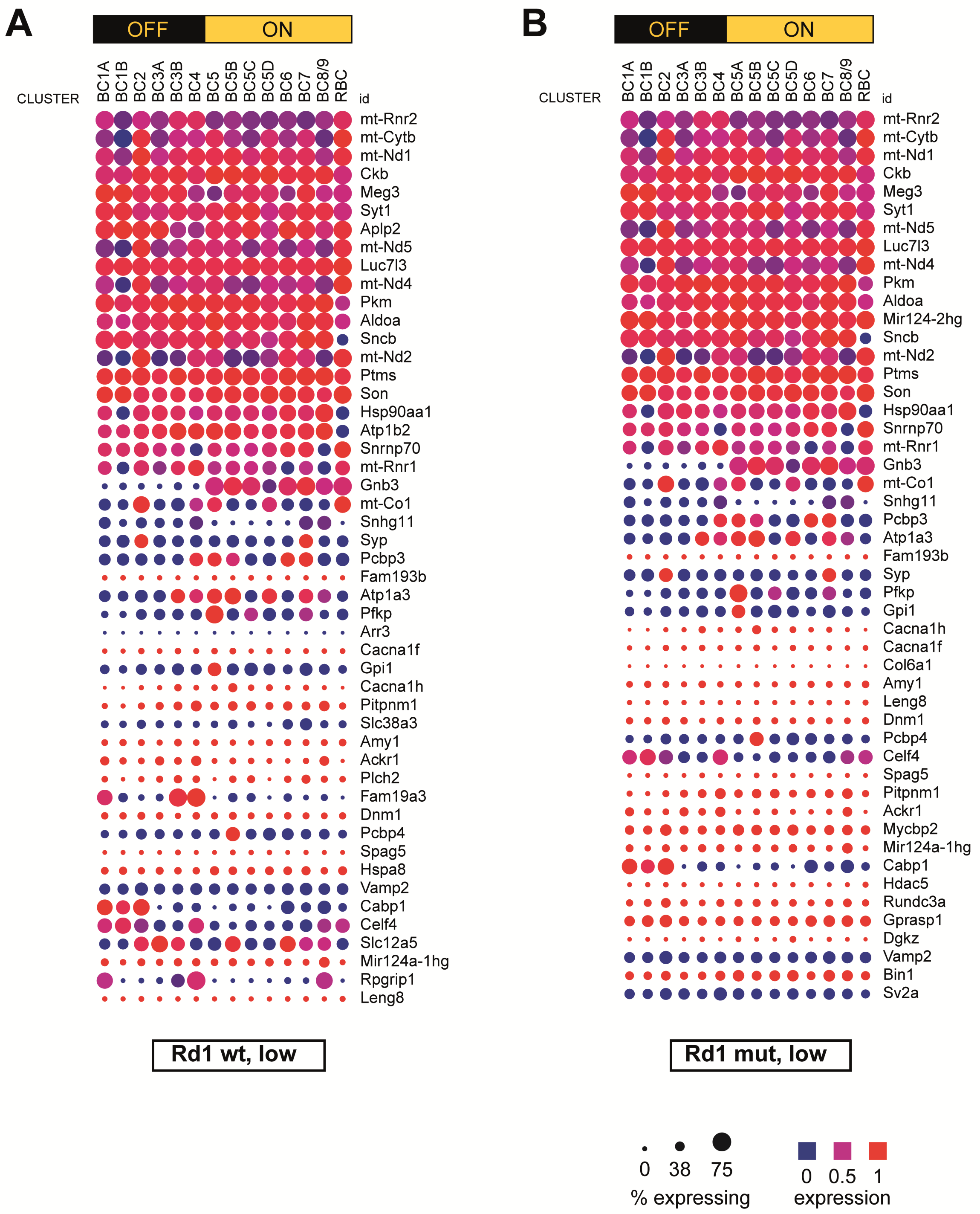

Figure S3. RNAseq data of the low populations in mice.
A, B) Dot plot illustration of gene expression in bipolar cells from Rd1 wt (A) and mut (B), following submission of the 50 genes of our RNAseq having the highest read counts on the Single Cell Portal, as seen in Figure 2. ON and OFF bipolar subtypes are shown, with the individual bipolar subtype cluster below. Note the random pattern of expression of most genes compared to the results from the high populations.

Supplemental Tables

Table S1: RNAseq data of mouse and NHP samples.

Table S2: Gene specific primers for mouse genes.

Gene	Forward 5' – 3'	Reverse 5' – 3'
Alas1 *NM_001291835.1*	GGATGGCGTCATGCCAAAAA	GTCGATCAGCAAACTCGTGC
Gapdh *NM_001289726.1*	AATGGTGAAGGTCGGTGTGAA	GCAACAATCTCCACTTTGCCA
Grm6 *NM_173372.2*	TGCACTACATCCGAGCAGTC	TGGAAGATGTCATAGCGCCC
Pcp2 *NM_001129803.1*	CAGAACCCAGAAAGCCAGGGT	GGGTGTTGACCAGCATATCCA
Prkca *NM_011101.3*	GACAAGCCGCCATTTCTGAC	TCCATGACGAAGTACAGCCG
Cabp5 *NM_013877.3*	GCTTGGTGAACACTACCTGCT	TCCTGTCCCAGTGGTCTCTC
Rho *NM_145383.2*	AGGGCTTCTTTGCCACACTT	GGAAGTTGCTCATCGGCTTG
Kcnip3 *NM_019789.4*	GGCTCAGACAGCAGTGACAGT	GGTCTGAGCTTGTAGCTGGTC
Tacr3 *NM_021382.6*	GTGACAAGTACCATGAGCAGC	CATGGTAGGGTAGCCAGCAG

Table S3: Gene specific primers for monkey/human genes.

Gene	Forward 5' – 3'	Reverse 5' – 3'
HPRT1 *NM_000194.2*	TTCTTTGCTGACCTGCTGGA	GTCCCCTGTTGACTGGTCATT
GRM6 *NM_000843.4*	TTCGAGCTGTCCGCTTCAA	ACTGGAAGATGTCGTACCGC
PCP2 *XM_005587767.2*	GGAGTCCAGGGACATGATGG	ACGTGGCTCAGCAGATTGAA
CABP5 *XM_005589722.2*	TTTGTGGAGCTGATGACCCC	GATCTCCCCATCTCCGTTCG
RHO *NM_001283360.1*	ACCCTCTACACCTCTCTGCAT	GACCACCAAGGACCACAGG
PRKCA *NM_002737.2*	CACGAGGTGAAGGACCACAA	CAAACTTGGCACTGGAAGCC
TACR3 *NM_001059.2*	TTTGTGCAATGGCCAGAAGG	CCATGATGAGCAATGGGAAACA
